# DILI_*C*_: An AI-Based Classifier to Search for Drug-Induced Liver Injury Literature

**DOI:** 10.3389/fgene.2022.867946

**Published:** 2022-06-29

**Authors:** Sanjay Rathee, Meabh MacMahon, Anika Liu, Nicholas M. Katritsis, Gehad Youssef, Woochang Hwang, Lilly Wollman, Namshik Han

**Affiliations:** ^1^ Milner Therapeutics Institute, University of Cambridge, Cambridge, United Kingdom; ^2^ LifeArc, Stevenage, United Kingdom; ^3^ Department of Chemistry, Centre for Molecular Informatics, University of Cambridge, Cambridge, United Kingdom; ^4^ Department of Chemical Engineering and Biotechnology, University of Cambridge, Cambridge, United Kingdom; ^5^ Cambridge Centre for AI in Medicine, University of Cambridge, Cambridge, United Kingdom

**Keywords:** drug-induced liver injury (DILI), natural language processing (NLP), machine learning (ML), artificial intelligence (AI), pattern mining, shiny app

## Abstract

Drug-induced liver injury (DILI) is a class of adverse drug reactions (ADR) that causes problems in both clinical and research settings. It is the most frequent cause of acute liver failure in the majority of Western countries and is a major cause of attrition of novel drug candidates. Manual trawling of the literature is the main route of deriving information on DILI from research studies. This makes it an inefficient process prone to human error. Therefore, an automatized AI model capable of retrieving DILI-related articles from the huge ocean of literature could be invaluable for the drug discovery community. In this study, we built an artificial intelligence (AI) model combining the power of natural language processing (NLP) and machine learning (ML) to address this problem. This model uses NLP to filter out meaningless text (e.g., stop words) and uses customized functions to extract relevant keywords such as singleton, pair, and triplet. These keywords are processed by an apriori pattern mining algorithm to extract relevant patterns which are used to estimate initial weightings for a ML classifier. Along with pattern importance and frequency, an FDA-approved drug list mentioning DILI adds extra confidence in classification. The combined power of these methods builds a DILI classifier (DILI_*C*_), with 94.91% cross-validation and 94.14% external validation accuracy. To make DILI_*C*_ as accessible as possible, including to researchers without coding experience, an R Shiny app capable of classifying single or multiple entries for DILI is developed to enhance ease of user experience and made available at https://researchmind.co.uk/diliclassifier/. Additionally, a GitHub link (https://github.com/sanjaysinghrathi/DILI-Classifier) for app source code and ISMB extended video talk (https://www.youtube.com/watch?v=j305yIVi_f8) are available as supplementary materials.

## 1 Introduction

Drug-induced liver injury (DILI) is a class of adverse drug reactions (ADR) which is an issue in both clinical and research settings. Although DILI can be mild, resolving once administration of the problem drug is discontinued, it lies on a spectrum and can also be severe. DILI is the most frequent cause of acute liver failure in the majority of Western countries (Hoofnagle and Björnsson, 2019) and is a major cause of attrition of novel drug candidates (Church and Watkins, 2018) and accounts for almost one-quarter of clinical drug failures (Watkins, 2011). As new findings on DILI are often published in the scientific literature, collating these data from the literature is useful for risk assessment during drug development and in the clinic. However, currently, manual trawling of text from the literature is the main route of obtaining relevant information about DILI from research studies. This is an inefficient process prone to human error, and modern computational techniques for mining textual data can improve it. A model capable of retrieving DILI-related articles from the huge ocean of literature could be invaluable for the drug discovery community. Natural language processing (NLP) involves using computational techniques to extract information and insights from text data. Previous studies have applied NLP techniques to identify the relevant literature for challenges in drug discovery, including the goal of drug repurposing (Zhu et al., 2020) and collating information on COVID-19 for researchers (Wang and Lo, 2021). There have been a small number of studies addressing this DILI in the literature problem to date. A collaboration between Pfizer and the Comparative Toxicogenomics Database (CTD) used text mining to aid manual curation to collate information from over 88,000 articles relating to 1,200 drugs and their links to several toxicities, including hepatotoxicity; although it is a valuable resource, it is limited to the articles and drugs it focused on (Davis et al., 2013). LimTox, a Web tool built in 2017, uses text mining to identify DILI events in the PubMed literature (available at the time of development) associated with drugs (Cañada et al., 2017). This is useful for a specific drug of interest but does not classify the literature itself as related to or unrelated to DILI and appears to not update the literature accessed, which would make it less useful for newer drugs (Cañada et al., 2017). A more recent study has applied AI-based NLP approaches to sentences contained within FDA drug labeling documents to classify them as suggesting DILI risk for the drug in question or not (Wu et al., 2021). Additionally, previous attempts have been made to classify adverse drug events using NLP on available data (Harpaz et al., 2014). Databases of drug side effects also contain DILI-related information (FDA (2021); Kuhn et al. (2016). In this study, NLP is used to extract relevant patterns from the literature, and this knowledge is combined with information related to DILI from publicly available databases. This combined information is used to train a classifier to classify the literature as DILI-related or not.

## 2 Workflow

**FIGURE 1:**
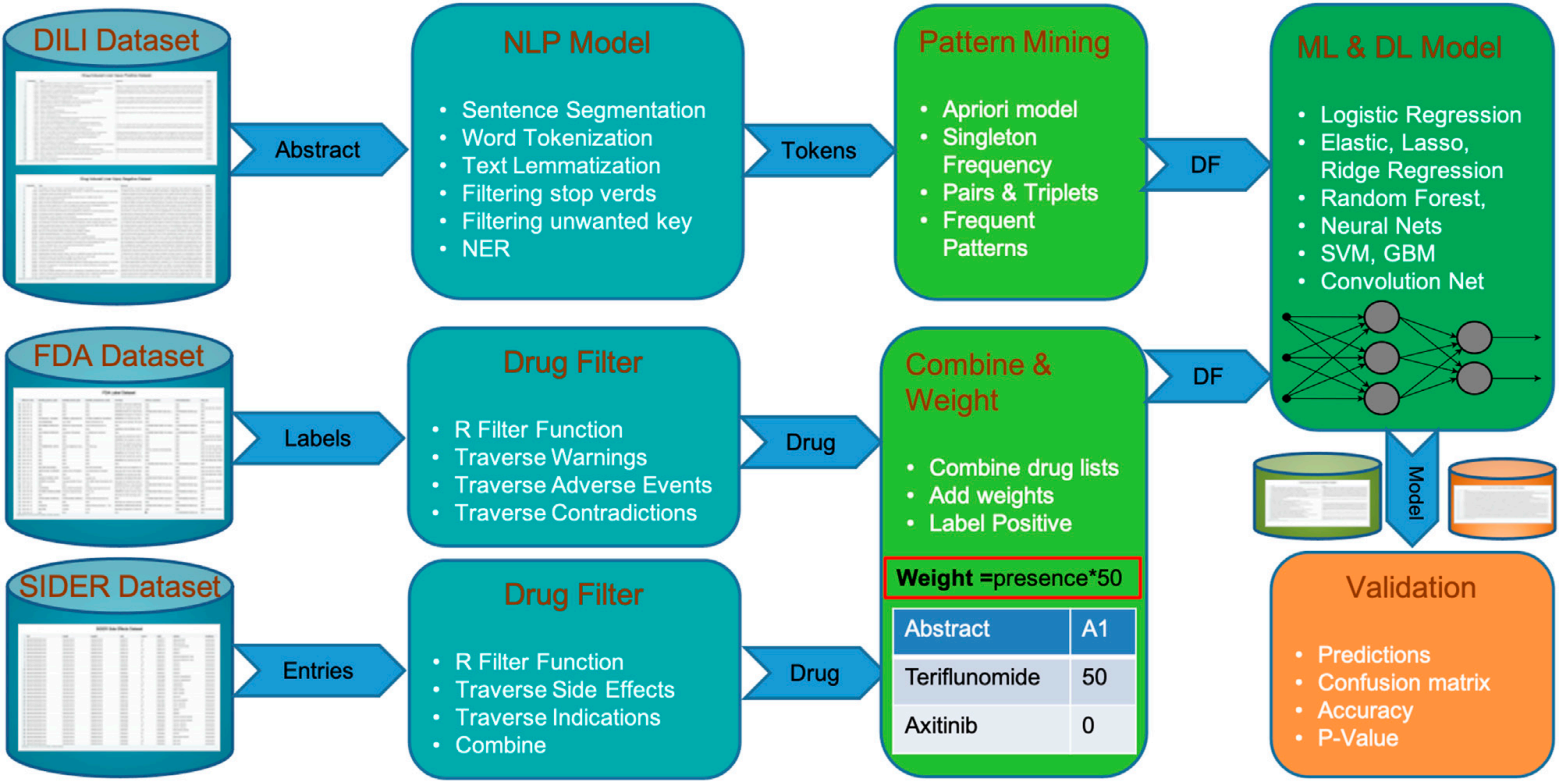
Steps of DILI_*C*_ from a dataset of DILI-positive and DILI-negative articles to validations showing integration of FDA and SIDER datasets.

The DILI_*C*_ pipeline can be subdivided into four phases, as shown in Figure 1. In phase I, a curated DILI dataset is uploaded, as well as information from external cohorts relevant to this classification task. The DILI dataset is a well-curated dataset that contains the literature labeled as positive (related to DILI) and negative (totally unrelated to DILI); this was obtained from the CAMDA team. This dataset, which contains a balanced cohort of DILI-positive and DILI-negative literature, was divided into a discovery and a validation set maintaining the positive and negative class split. Along with this curated DILI dataset, our pipeline is enriched with information from FDA and SIDER adverse event datasets, where DILI is mentioned as a side effect.

In phase II, the DILI_*C*_ pipeline processes both these internal (curated DILI literature) and external (side effect datasets) cohorts to extract relevant information. This extraction process is quite simple and straightforward for the external cohort. This process examines database annotation related to adverse events for each drug and where DILI is mentioned as a side effect, the drug’s generic information (generic name, brand name, and compound) is retained. On the other hand, the extraction process for the internal cohort is more complicated and computationally intensive. This process combines several standard natural language processing tasks such as sentence segmentation, word tokenization, text lemmatization, and filtering (i.e., stop words and unwanted key), with customized token generation of word sets of varying lengths (single word, pair, triplets, etc.). A list of named entities with multiple lengths is stored for pattern mining and scoring in the next phase.

Phase III concentrates on pattern mining and scoring these mined patterns. This step uses the distributed apriori algorithm Rathee et al. (2015) to extract superset patterns, which occur frequently, and scores them based on their length, frequency, and whether they appear in the positive and negative classes. The overall score of a pattern is calculated by its score in the positive or negative class divided by the total score in both classes. The scoring gives higher weight to the names of drugs coming from FDA and SIDER external cohorts than to patterns extracted *via* text mining because these drugs have been associated with DILI in highly trusted databases. Finally, all the patterns with their score for each abstract are stored in a matrix format, which can be fed to any ML classifier.

In phase IV, a scoring matrix is utilized by multiple machine learning classifiers to learn and predict labels for validation data. Instead of using a favorite ML classifier, our pipeline feeds the score matrix to multiple models (i.e., logistic regression, elastic net (lasso and ridge), random forest, neural nets, support vector machines, and gradient boosting machine) to find out which models suit our dataset based on cross-validation accuracy. The best classifier with the highest cross-validation accuracy is used to make predictions on unseen validation cohorts. The validation process is extended with another unbalanced validation cohort in the second phase.

The DILI_*C*_ pipeline is capable of classifying the literature as relevant or irrelevant for DILI studies with high-accuracy measures. It can be easily adapted for any other adverse event. A detailed description to build this pipeline is available in the Methods section.

## 3 Materials and Methods

We built an artificial intelligence (AI) model combining the power of natural language processing (NLP) and machine learning (ML) to extract the relevant literature for DILI from the ocean of published articles. This model combines the information available in the title and abstracts of scientific articles with information from external databases to improve efficacy and accuracy. A detailed procedure is available in Algorithm 1, which contains all the steps to build this model.

Algorithm 1 Classify the Literature as DILI-Positive or DILI-Negative.

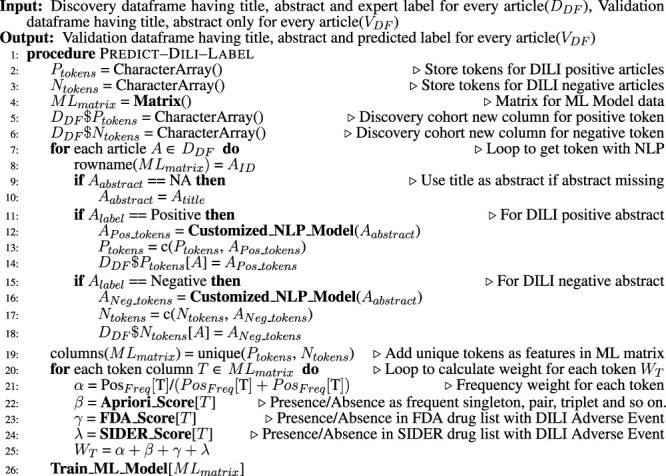

### 3.1 Data Preparation

A well-curated dataset of ˜28,000 DILI-annotated articles was obtained from the CAMDA team CAMDA (2021). This dataset was generated after filtering out the most obvious DILI literature, which makes the task of classification challenging, but more representative of the challenge of sorting through the real-world literature beyond the obviously DILI-related or entirely unrelated articles. All the articles in this dataset are labeled as DILI-related (DILI positives) or not related to DILI (DILI negatives) by an experienced panel of experts. We used approximately half of these data with a balanced split of DILI-positive and -negative to extract insights and train a model (discovery set). The remaining half was kept as a validation set.

We divided the discovery set of 14,203 articles into training (80%) and testing (20%) sets, consistent with their labels. Overall, we used 5,741 DILI-positive and 5,620 DILI-negative as a training set and 1,436 DILI-positive and 1,406 DILI-negative as a test set.

### 3.2 Natural Language Processing Model

An NLP model with some customization was used to extract the relevant information from the available training cohort (Algorithm 2). It starts with the most basic NLP step sentence tokenization on titles and abstracts, followed by word tokenization. A customized word tokenization method was developed to generate keyword sets of singleton, pairs, triplets, and so on. This step generates combinations containing only nouns and adjectives and filters out irrelevant text like stop words using the R UDPipe package (Straka and Straková, 2017). These keyword sets were processed for text lemmatization and stemming to generalize the list. The output of this NLP model was a vector containing all keyword sets as features, and for each of these, their frequency and length (singleton and pair) were stored as weights for pattern mining. This NLP model was applied to both titles and abstracts.

Algorithm 2 Customized NLP Model to Extract Tokens From Abstract.

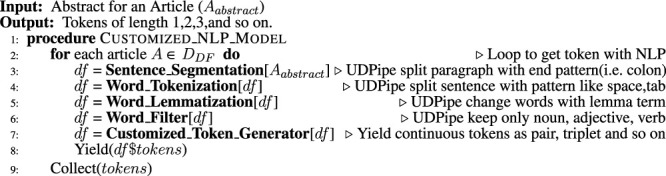

### 3.3 Pattern Mining

Along with the total frequency of a keyword set, the frequency of the keyword and its subsets in terms of the number of articles (DILI positive or DILI negative) in which it appears was calculated. The pattern mining ML algorithm apriori was used for this. In this way, we included the frequency of a keyword set and its subset as a factor for weighting that keyword set. A distributed processing-based implementation of apriori was used to minimize the overall processing time.

Algorithm 3 Add score for presence/absence in external cohort FDA and SIDER.

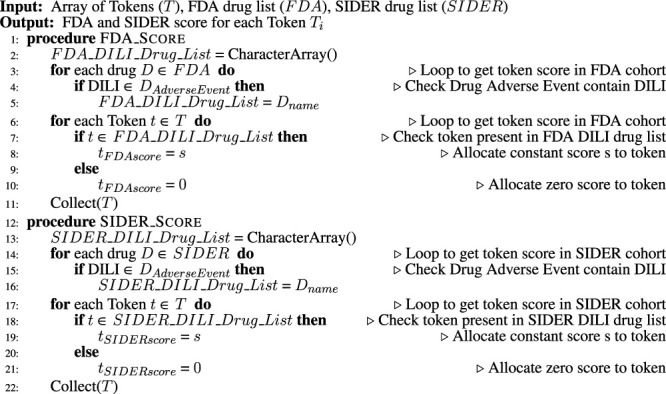

### 3.4 External Cohort Integration

Since external datasets contain information that could be advantageous in classifying the DILI literature, two were integrated into the model. These two publically available datasets were the FDA-approved drug list (FDA, 2021) and SIDER adverse events dataset (Kuhn et al., 2016). From these two datasets (Algorithm 3), a list of drugs with DILI as adverse events or warning was extracted, and these drugs were given a higher weight than others without such warnings. The side effects field of the SIDER database for drugs was helpful to add extra information to this highly weighted list.

### 3.5 Classifier

The final vector of keywords, along with their updated weights, was given as input to various well-known ML and AI models (logistic regression, elastic net, random forest, neural net, support vector machine, gradient boosting machine, convolution neural networks, and LSTM) to train a classifier. The weight of a keyword was calculated by its total frequency, length, FDA, and SIDER list presence or absence.

$$W_{T}=\overset{j}{\underset{i=1}{\sum }}W_{fi}*Key_{i}+\overset{j}{\underset{i=1}{\sum }}W_{li}*Key_{i}+\overset{j}{\underset{i=1}{\sum }}W_{fdai}*Key_{i}+\overset{j}{\underset{i=1}{\sum }}W_{sideri}*Key_{i},$$ (1)

where *W*_*T*_ represents the total weight for a study, the *key* represents the weight for presence (1) or absence (0) of a keyword set, (*W*_*f*_) represents the weight for frequency of a keyword set, *W*_*l*_ represents the weight for length of a keyword set (for instance singleton 1, pair 2, and triplet 3), *W*_*fda*_ represents the weight for the presence and absence in the FDA list with the DILI adverse event, and *W*_*sider*_ represents the weight for the presence and absence in the SIDER list with DILI adverse events.

All ML classifiers are trained on an 80% split (11,361 abstracts) of the discovery set. The classifier with the highest cross-validation accuracy (gradient boosting machines) was tested on a standout test set (20% split with 2,841 abstracts) and external validation cohort (14,000 abstracts). Table 1 shows the confusion matrix for the standout (20%) testing set.

**TABLE 1:** Confusion matrix of GBM classifier applied to standout abstract cohort.

		True class	
		Positive	Negative
Predicted class	Positive	1335	44
	Negative	101	1362

**FIGURE 2:**
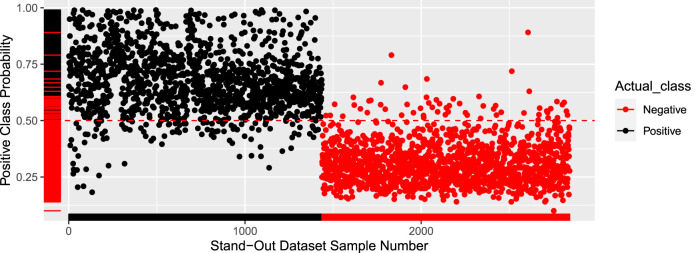
Prediction probability plot.

The results on the external validation set were also quite promising, with an accuracy of 94.89%. The model was iterated 10 times with different test sets to get the average accuracy of 94.9%. Figure 2 shows the probability of every sample being positive. Any sample with a probability higher than 50% is labeled as DILI positive. The cutoff of 50% can be adjusted to closely reflect a real-world dataset that will have far more negative pieces of literature.

## 4 Results

**FIGURE 3:**
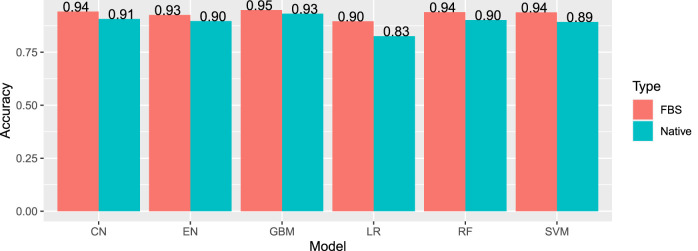
Internal accuracies for all ML classifiers (EN, elastic net; LR, logistic regression; SVM, support vector machines; CN, convolution network; RF, random forest; GBM, gradient boosting machine; FSB, feature selection-based model) showing that GBM has the highest accuracy.

The most effective model was gradient boosting machines (Figure 3), with 94.76% accuracy, when applied to the internal hold out test set of 2,842 articles, half of which were DILI-positive and half DILI-negative. The inclusion of the FDA and SIDER datasets improved the accuracy of the GBM model in the validation set and on an additional external set (Table 2). The final model is used to predict the labels for the external validation cohort shared by CAMDA. We got encouraging results with an accuracy of 94.14% and F1-score 94.08%. The highlight of the model was its recall value of 96.02%.

**TABLE 2:** Results for the GBM model applied to the validation set and additional external sets of DILI and the non-DILI literature. The inclusion of FDA and SIDER datasets improved the GBM model.

	Validation set (14211)				Additional external set (2000)			
	Accuracy	F1 score	Recall	Precision	Accuracy	F1 score	Recall	Precision
GBM (abstract only)	0.9386	0.9376	0.9631	0.9133	0.8845	0.8936	0.9700	0.8284
GBM (+FDA)	0.9406	0.9396	0.9659	0.9147	0.8915	0.8992	0.9680	0.8395
GBM (+SIDER)	0.9414	0.9408	0.9602	0.9221	0.9025	0.9094	0.9790	0.8491

DILI_*C*_ was then applied to an unseen additional external set, which was an unbalanced DILI cohort, making it more reflective of real-world data. On the additional external set, accuracy was 90.25% and an F1-score of 90.94%. The recall value was improved with this set, with a value of 97.9%.

## 5 Discussion

DILI_*C*_ is a model with high accuracy which is useful to the community to classify the literature as related to or unrelated to DILI, which can help perform DILI risk assessment for drugs during development, repurposing, or in the clinic. Although it was developed to classify the DILI literature, it has been designed to handle any adverse event classification problem, so it has applications for drug risk assessment beyond just liver injury to toxicities in other tissues. We note that complex machine learning AI models are known to have the power to magnify weak signals.

In order to minimize the pressure on ML models and reduce the risk of such erroneous magnification, during the development of DILI_*C*_, a strong focus was placed on the data cleaning and processing steps of the model. Another potential issue is the chance that the inclusion of the SIDER dataset could introduce bias against publications relating to drugs that are not yet included therein. Reassuringly, even without the inclusion of this database, DILI_*C*_ performs well, with an accuracy of 94.06% on the validation set and 89.15% on the additional external set. There is still potential to improve DILI_*C*_ in the future. Later steps like customized word segmentation, pattern mining, and external relevant cohorts add power to DILI_*C*_, and there is still plenty of scope to adjust the weights for these steps. In addition, as other databases related to drug toxicity and side effects are developed, these could be integrated to improve the model. To make DILI_*C*_ as accessible as possible, including researchers without coding experience, the R Shiny app capable of classifying single or multiple abstracts for DILI is developed to enhance ease of user experience and made available at https://researchmind.co.uk/diliclassifier/.

## 6 Conclusion and Future Work

DILI_*C*_ is a novel tool to classify the literature as related to DILI or not. This is significant as it has the potential to aid researchers in drug development and research and clinical settings during the risk assessment. DILI_*C*_ is implemented in such a way that it can be modified to classify any other drug’s adverse reactions and is not limited to DILI. Therefore, the DILI_*C*_ code available at the GitHub link could be useful for researchers interested in drug-induced neural, cardiovascular, or renal toxicities for example. The Shiny app for DILI_*C*_ provides the tool in a user-friendly and accessible way that can be easily used by nonprogrammers who have the literature they want to classify. Additionally, an ISMB extended video talk is available as a supplementary resource that explains the pipeline step by step (https://www.youtube.com/watch?v=j305yIVi_f8).

Work is ongoing to improve the classification accuracy measures of DILI_*C*_ by increasing weights for well-known entities like genes, drugs, species, and pathways. These entities will be extracted using transformers trained on PubMed data (i.e., BioBERT (Lee et al., 2020)). We are also working to add more features to our app so that it will be able to take any adverse event and its literature as an input to classify the related literature.

## Data Availability

The datasets presented in this study can be found in online repositories. The names of the repository/repositories and accession number(s) can be found at: http://camda2021.bioinf.jku.at/doku.php/contest_dataset#literature_ai_for_drug_induced_liver_injury.
